# Product Assembly Assistance System Based on Pick-To-Light and Computer Vision Technology

**DOI:** 10.3390/s22249769

**Published:** 2022-12-13

**Authors:** Darko Hercog, Primož Bencak, Uroš Vincetič, Tone Lerher

**Affiliations:** 1 Faculty of Electrical Engineering and Computer Science, University of Maribor, Koroška Cesta 46, SI-2000 Maribor, Slovenia; 2 Faculty of Logistics, University of Maribor, Mariborska Cesta 7, SI-3000 Celje, Slovenia; 3 Faculty of Mechanical Engineering, University of Maribor, Smetanova ulica 17, SI-2000 Maribor, Slovenia

**Keywords:** product assembly, pick-to-light (PTL), computer vision, LabVIEW, performance analysis

## Abstract

Product assembly is often one of the last steps in the production process. Product assembly is often carried out by workers (assemblers) rather than robots, as it is generally challenging to adapt automation to any product. When assembling complex products, it can take a long time before the assembler masters all the steps and can assemble the product independently. Training time has no added value; therefore, it should be reduced as much as possible. This paper presents a custom-developed system that enables the guided assembly of complex and diverse products using modern technologies. The system is based on pick-to-light (PTL) modules, used primarily in logistics as an additional aid in the order picking process, and Computer Vision technology. The designed system includes a personal computer (PC), several custom-developed PTL modules and a USB camera. The PC with a touchscreen visualizes the assembly process and allows the assembler to interact with the system. The developed PC application guides the operator through the assembly process by showing all the necessary assembly steps and parts. Two-step verification is used to ensure that the correct part is picked out of the bin, first by checking that the correct pushbutton on the PTL module has been pressed and second by using a camera with a Computer Vision algorithm. The paper is supported by a use case demonstrating that the proposed system reduces the assembly time of the used product. The presented solution is scalable and flexible as it can be easily adapted to show the assembly steps of another product.

## 1. Introduction

The Industry 4.0 (I4.0) concept is taking over classical paradigms that once fuelled the advancements in manufacturing processes. Industry 4.0 was coined to describe the immediate need for economic, sociological, and political changes. Those necessities stem from end-users, who are the drivers of the industry, as short product development periods, individualization on demand, flexibility, decentralization and resource efficiency are sought after [1].

The I4.0 concept includes mainly enabling technologies, such as Cyber-Physical Systems (CPS), Internet of Things (IoT) and cloud computing [2]. The first one describes the ability to intertwine the physical properties of a system coupled with advanced computational algorithms. A well-known example is the field of Predictive Maintenance [3], which informs the user of an impending service. The IoT relates to the interconnection of various devices that rely on sensory, communication, networking, and information processing technologies [2]. Sensor networks can gather data regarding the manufacturing process and then later use that data to optimize the process. Cloud computing refers to sharing documents, servitization, collaboration, distributed production and resource optimization [4]. The main advantage of cloud computing is the scalability of resources, which means that extra computing power is ensured if additional demand arises [5].

Simultaneously with the requirements that drive the I4.0 concept, a need for more advanced logistics systems has arisen to cope with the rising trends of E-commerce [6] and the concerning trends of workforce deficiency and ageing [7]. Those challenges can be overcome by introducing more advanced warehousing systems and methods along with (robot) assisted Order-Picking (OP) systems. Hence, the Logistics 4.0 (L4.0) term has been coined based on the Industry 4.0 term [8,9]. Logistics 4.0 describes advanced usage of various technological advancements such as those in Industry 4.0, such as smart devices, wearables, IoT and other CPS, which aim to reorganize some of the basic concepts of Logistics [9].

While both the I4.0 and L4.0 concepts aim toward reducing physical work and increasing automation, that is not always possible. In industrial environments, as in logistics processes, manual labour remains ever-present. This holds especially true for processes that cannot be fully automated for various reasons, e.g., over-complexity or costs of the automated solution, items that require a lot of customization, custom-crafted (hand-made) products, etc. In this case, the quality of products or services is directly dependent on assembly workers or order-pickers. Therefore, there is a need to create a multi-skilled workforce capable of performing multiple tasks, or invest in worker-assisted systems [10]. By investing in enabling technologies, not only does worker productivity and product quality rise, but ergonomics can also be improved significantly [11]. Assembly workers and order-pickers are exposed daily to the dangers of work-related musculoskeletal disorders (MSD) and back health problems [12].

Cohen et al. [13] noted that considerable efficiency improvements could be achieved by introducing I4.0 concepts into manual assembly stations. In their work, they have implemented an Assembly System 4.0 framework based on I4.0 concepts on a multi-model batch production flow line for industrial refrigerator manufacturers. They follow four main I4.0 principles: connectivity, information, knowledge and smartness. They note that the Self-Adapting Smart System (SASS), adapting using acquired information during the operation coupled with continuous support to the operators, will increase flexibility, agility, scalability, and productivity significantly.

A systematic literature review on the topic of worker assistance systems in manufacturing has been presented by Mark et al. [14]. The authors define worker assistance systems as “technical systems that support the worker during manufacturing or assembly tasks without replacing him, without overruling him and without posing any danger to the worker”. They divide the existing literature on assistance systems into three categories: (1) Sensorial, which extends the sensing capabilities; (2) Physical, which extends physical capabilities; and (3) Cognitive, which extends cognitive capabilities such as “orient” or “decide”. In the sense of aiding assembly processes in manual assembly workstations, the most used systems are various head-mounted devices (augmented and mixed reality [15]), tablets [16], various projection systems [17] and motion sensing devices [18]. The information regarding assembly processes can be displayed in the form of animation, graphic notes, speech, etc. In addition, exoskeletons [19] and robotic co-workers [20] can be introduced to reduce the workload on workers; however, this exceeds the scope of this paper.

The scientific community is studying augmented reality applications in aiding assembly workers thoroughly, as they are proving very prominent in the area [21]. Moghaddam et al. [22] showed that AR has recently been adopted as a novel experimental training technology for faster training and upskilling manufacturing workers, potentially reducing new hire training time by 50%. Furthermore, they note that error reduction by using the AR is also sustained after the AR support is removed. However, by introducing highly technological assistance systems into workplaces, one cannot expect an instant rise of productivity and error reduction, as user acceptance is crucial for the diffusion of new technologies and working environments [23]. Furthermore, presenting information through AR plays an important role, as Wang et al. [24] noted that by unintuitive expression of information, the guided assembly efficiency may be low, and the rate of operation error may be high. In a survey by Minow et al. [25], which included 25 young people, who are not trained assembly workers, they found that the pick-to-light (PTL) system proved to be more helpful than an AR-aided solution.

Additionally, to improve workplace ergonomics and reduce the cognitive load, the effects of using AR on the eyes are usually neglected, along with the fact, that AR glasses are less or not appropriate for people who are already wearing prescription glasses. Marklin et al. [26] noted that the decreased eye blink rate by using AR could lead to eye strain if used for prolonged periods without rest. Furthermore, wearing additional head devices for prolonged periods may cause neck fatigue. In that case, alternatives should be considered that do not require wearing additional hardware, such as PTL systems.

In intralogistics, and especially order-picking, there has been much effort to guide or automate the order-picking process, as it represents the most labour and time-intensive process in warehousing [26]. An order-picker travels around the warehouse, collecting (picking) the items specified in the order. Interestingly, an order-picking process is similar to the assembly process, where both the order-picker and assembler must follow instructions to complete an order or a product. Furthermore, both processes suffer from similar problems—ergonomics, questions regarding productivity, error prevention and detection.

Winkelhaus et al. [27] prepared a systematic review of existing order-picking techniques, comparing them by the level of automation for support and by the level of automation for substitution. Based on the I4.0 requirements, they conceptualized an Order-Picking 4.0 concept, which according to their definition, is a sociotechnical order picking system in which individual and heterogeneous customer orders are compiled efficiently and sustainably in small batch sizes from a large variety of goods in a warehouse. Thereby, Order Picking 4.0 considers high levels of automation of supportive and substitutive technologies, as well as human factor objectives. Based on that premise, Setayesh et al. [28] studied human order-picker factors extensively, since OP is still mainly a manual process. They identified distinct failure modes that may contribute to picking errors, such as vision, hearing, complexity, skills, memory demand, mental fatigue, physical fatigue, physical workload, motivation, supervision, and communication.

Since the earliest orders for order-pickers were paper printed, the OP relied solely on his previous experience and memory of the warehouse layout. Today, various paperless technologies are implemented to reduce cognitive load and assist the order-picker, such as a barcode/RFID scanner, voice-control, pick-to-light (pick-by-light), augmented reality (AR), head-mounted displays (HMD), projection, etc. A paperless order picking system consists of devices designed to facilitate the work of operators, mainly in terms of getting information on the product to be picked and its storage location [29]. Fager et al. [30] compared the impact of four different picking information systems (pick-by-paper, pick-by-light, pick-by-voice and pick-by-HUD) for kit preparation and concluded that in a single-kit preparation, a PTL system was associated with the highest time efficiency of the four studied systems.

In a PTL system, operators are guided by the lights installed on the warehouse shelves. For each micro-location, a light turns on, corresponding to the order picker’s order list. A button must be pressed to complete a single pick, and, in some cases, the barcode must be scanned. The system is also capable of handling multiple order-pickers using different coloured lights. A traditional PTL system can be upgraded with RFID, where the button no longer must be pressed, but instead, the RFID tag must be read with the RFID scanner. According to Allesandro et al. [31], the RFID reader can be mounted on the order picker’s glove, which means that both hands are free for picking. A PTL system is not necessarily stationary, as Su et al. [32] presented a robotic-based PTL system, which is operated based on the mutual behaviour analysis of human and mobile robots. Their PTL system is mounted on KIVA inventory pods, which are storage racks that can be moved around using KIVA mobile robots. According to Battini et al. [33], the main advantage of the PTL order-picking system is that only two types of technical errors can occur, compared to four in handheld barcode and RFID order-picking: (E1) Wrong item picked, but correct item confirmed error, and (E2) Wrong quantity picked error. In the RFID enhanced PTL application, (E2) can also occur, yet (E1) cannot, but an (E3) Wrong item picked, and the wrong item confirmed error can occur.

AR is also gaining the attention in OP applications, where its task is to convert logical information into visual guidance for an order-picker [34]. The order-picker must wear specialized glasses, which leaves both hands free for order-picking. Despite its time efficiency picking accuracy and low chance of error, AR order picking is prone to tracking lost in the industrial scene, where there are no distinctive landmarks for orientation.

All those support systems cannot automate the order-picking or assembly processes yet help substantially, as they provide more information on the micro-location of the product to be picked or provide information for the product to be assembled. Furthermore, the initial training period can be reduced significantly, as the OP process is guided independently of the pre-knowledge of the system. The proposed paper attempts to combine PTL technology used in order-picking with a manual assembly workstation to increase worker productivity and reduce the possibility of errors.

Inspired by the PTL method in the order-picking process, a prototype of an assembly assistance system based on PTL modules is presented in this paper. The proposed system guides the operator through the assembly process using a PC application and several PTL modules. A single PTL module is used for each assembly part. The PTL module consists of a microcontroller with an integrated Wi-Fi interface, a liquid crystal display (LCD) for displaying the current assembly component micro-location and quantity, and a pushbutton for acknowledging the picked object. The application on the PC guides the operator through the assembly process by showing all the necessary assembly steps and parts. Two-step verification is used to ensure that the correct part is picked out of the bin, first by checking that the correct pushbutton on the PTL module has been pressed and second by using a camera with a computer vision algorithm. The presented solution is adaptable in terms of needing multiple PTL modules for assembly operations that require a higher or lower number of assembly parts.

This paper is organized as follows. Related works are presented and analysed in Section 2. Section 3 presents the software and hardware setup of the proposed PTL-based assembly assistance system. Section 4 demonstrates the use of the system by giving an example of the assembly process of the Gillux-Puzzle mind game and some experimental results obtained using volunteer assemblers. Some pointers to future work are provided in the conclusion.

## 2. Related Works

The following section presents and analyses the closest related works from advanced manual assembly stations coupled with various assistive systems.

Bortolini et al. [35] proposed a Self-Adaptive Smart Assembly System (SASAS) capable of improving worker ergonomics by using two motion axes to position an easy-access fast-picking area for the fast-moving parts and a third axis for the reconfiguration of the working area height. The system has been tested in a full-scale assembly of an industrial chiller, showing that the SASAS prototype reduced the assembly cycle time and operator movements during the assembly process. This led to an increase in productivity by up to 70%. Even though the user still relies on his/her own assembly knowledge, the SASAS shows that considerable improvements can be made by reorganizing the assembly station’s layout. In [36], the authors further enhanced the original SASAS prototype and proposed a general framework guiding toward A3s (adaptive automation assembly systems) effective design and validation. The enhanced SASAS enables real-time reconfiguration based on the current assembly process. This is performed by using servosystems operated with a MATLAB-based GUI. Additionally, a motion analysis system has been employed to evaluate user ergonomics. In their second study, up to 38.6% reduction of assembly cycle time has been achieved with an improvement in the ergonomic work condition (REBA index reduction) of up to 15%. Turk et al. [10] presented a self-configurable assembly workstation using I4.0 technologies. The workstation is controlled by a smart algorithm that controls the smart workstation, enabling technologies and digital instructions for assembly tasks. Their smart assembly station is self-configurable and answers to the needs of individual ergonomic worker requirements, according to gender and body height (constitution). The assembly instructions are displayed via LPM software and pick-by-light technology (laser pointer), showing the correct section of the bin where the current part is stored. The workbench’s height, lighting and grab bins with parts and rotation of the assembly nest are controlled using a smart algorithm. Due to the simple usage of the system, thorough training pre-operation is not necessary. In a laboratory experiment, they compared the traditional assembly workstation and their smart manual assembly workstation for two different sample products made from LEGOs and evaluated the ergonomics of both systems. They concluded that the number of errors has been reduced by 72%, and while the overall assembly time in SAW was lower than in traditional WS, they have shown that the SMAW instruction program issued instruction slower than the worker’s skills permitted. The ergonomics score was much higher than those in TWS, since the smart algorithm optimizes the layout of the worktable. Yin et al. [37] presented an interaction-free assembly assistance tool (SARAMS) that monitors an operators’ hand activity and process completeness, to recognize the assembly state and provide the operator with instructions accordingly. Users are only issued with the augmented contents when they get stuck or go wrong in an assembly process. An assembly process completeness inspection is based on image feature matching. A prototype system requires only a head-worn device, freeing both hands for the assembly. The system has been evaluated on a 15-part product. The authors concluded that the proposed system had realized all its desired properties, including automatic monitoring, adaptive assistance, higher interaction efficiency and integrated hardware setup. Lai et al. [38] proposed a smart instruction system with the support of AR and deep learning-based tool detection, which is intended to improve the worker’s performance through assistive instructions (texts, videos, 3D animations). The developed system consists of multi-modal AR instructions and a tool detector. The hardware portion of the system comprises two cameras, used to capture the worktable surface from two perspectives to prevent a mix-up of tools and parts in addition to AR glasses worn by the assembly worker. The tool detector was developed using a Faster R-CNN model trained on a CAD-based synthetic tool dataset, which detects real physical tools with an average precision of 84.7%. It is used to prevent misusing assembly tools. The proposed system performance is evaluated on a CNC carving machine assembly by comparing the manual assembly instructions to the instructions issued by the AR. The manual and the proposed solution are compared in terms of completion time and assembly errors, which show a 33.2% and 32.4% reduction, respectively. Wang et al. [39] proposed a novel platform for remote collaboration based on AR, designed to assist industrial assembly tasks remotely. A remote expert sees the AR replica of the worktable plane, whilst the worker is presented with visual clues from the remote expert. They compare two methods of presenting the helpful information to the assembly worker: sharing AR annotations and Gaze Control (GC). The first refers to drawing shapes around the object that requires interaction, while the second refers to sharing a remote expert’s head pointer. The experiments showed that the GC is a superior guidance option since users are pointed to a certain point more clearly than by AR annotations. Ojer et al. [40] have developed a projection-based AR assistance system for manual Printed Circuit Board (PCB) assembly, which consist of an illumination system, a 2D high-resolution image acquisition setup a screen and a projector. No AR glasses are needed, which reduces worker eye strain, yet the illumination of the worktable area must ensure that the light from the projector remains predominant. Their system supports dynamic projection of the instructions, updated in real-time, as well as performing the verification of the operator’s current operation. Since the instructions are simple, they are presented in the form of rectangle bounds around the components to be assembled in the next step. The system showed that compared to the original PCB assembly procedure, less errors occurred, especially when workers are faced with a new PCB type. Sorostinean et al. [41] presented an extensive assembly assistance system, which consists of several assistive technologies, such as an object and hand movement sensor, eye tracking and GSR biosensor, a posture and facial expression sensor and a large touchscreen embedded in the tabletop. The AAS tabletop is divided into an assembly area, a components storage area and a video instruction display area. Additionally, the user is presented with voice instructions and user interaction buttons, which allow the user to play/repeat instructions or go to the previous or next instruction. In this work, they extend their previous contributions with a system state predictor based on decision trees with ensemble learning that can provide support via adaptive instruction considering the current assembly progress. The prediction is based on past assembly processes of a certain product, where a model is used to determine the most likely next assembly step, given the current assembly stage. The goal of a state predictor is to support inexperienced workers in their pre-operation stage without a human trainer and assist experienced workers in the manufacturing process. Their results have shown that ensemble learning with decision tree components is best suited for adaptive assembly support systems.

## 3. Materials and Methods

### 3.1. The Architecture of a PTL-Based Assembly Assistance System

**Figure 1:**
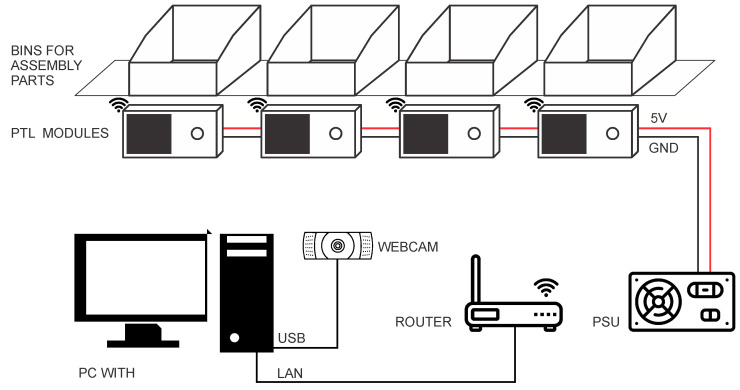
System architecture.

The developed system includes (Figure 1): (1) A PC with a touch screen monitor, (2) A USB camera, (3) PTL modules, (4) Bins for assembly parts, (5) A router, and (6) A power supply unit (PSU) for PTL modules. A touchscreen monitor (1920 × 1080, 21.5 inches) is used for interaction between the assembler and the assembly system. The touchscreen provides the assembler with a quick selection of the buttons available in the Graphical User Interface (GUI). The camera, used for part detection, is connected to the PC using a USB serial bus.

The router creates a local wireless network to which all used PTL modules connect at startup. Above each PTL module is a plastic bin for assembly parts. Each PTL module includes a liquid crystal display (LCD) and a user button. The PTL modules are connected to a 5 V power supply voltage.

The main application, which runs on a PC, guides the assembler through the assembly process. The program shows the individual steps of the assembly process in a Graphical User Interface and handles the communication with the PTL modules. The communication is realized using the TCP/IP protocol. The main application has the role of master, while all PTL modules operate as slaves. The master program establishes the TCP/IP communication with the selected PTL module, sends a message to it and waits for the response. The main application can set a new value on the PTL module’s LCD or check the currently displayed value.

When the new assembly step is displayed on the touchscreen monitor, the operator must take one or more elements from the corresponding bin. The number of parts needed in the assembly step is displayed on the LCD of the PTL module. The operator must confirm each element taken out of the plastic bin by pressing a button on the corresponding PTL module.

### 3.2. Hardware

#### 3.2.1. The Workbench

**Figure 2:**
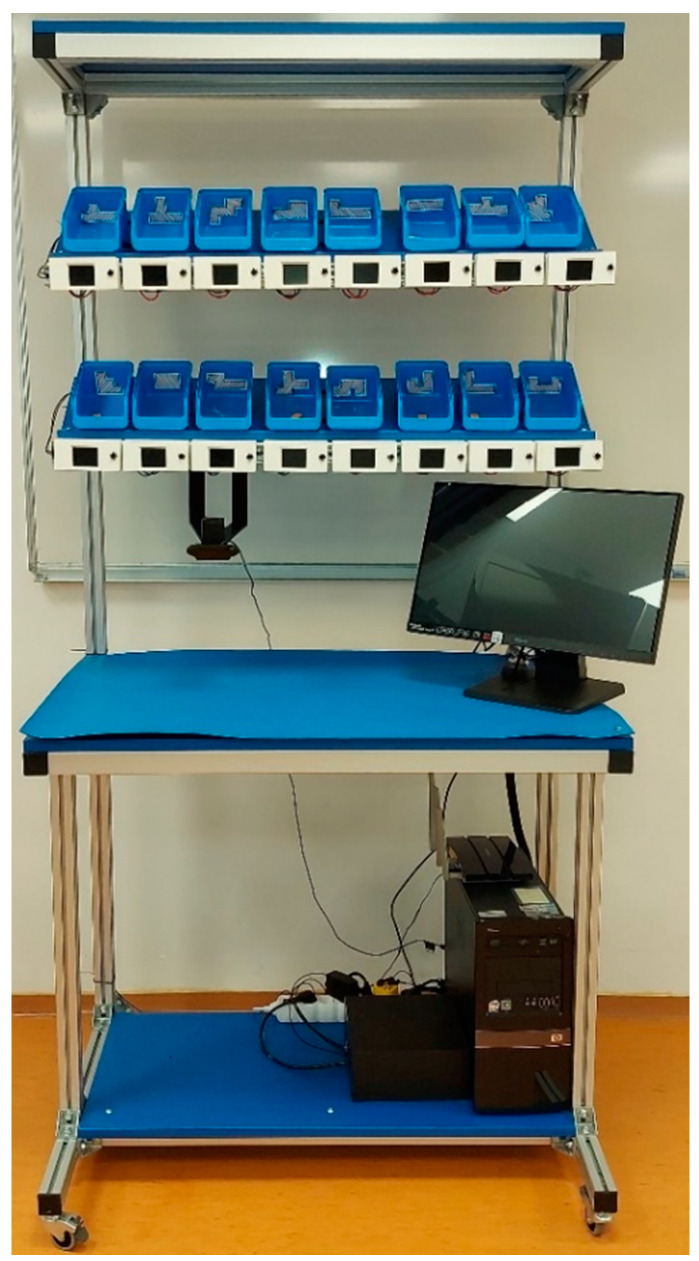
The workbench.

The workbench (Figure 2) is made of 40 mm × 40 mm aluminium profiles and is placed on wheels to make it easier to move around the room. The work surface of the table measures 1000 mm × 600 mm and is covered by an ESD (Electrostatic discharge) pad. Above the working surface are two shelves on which the plastic bins and PTL modules are placed. The shelves are angled, to make it easier for the operator to reach the components placed in the bins. At the front of the shelves is a DIN rail used to attach the PTL modules.

#### 3.2.2. Pick-To-Light Modules

**Figure 3:**
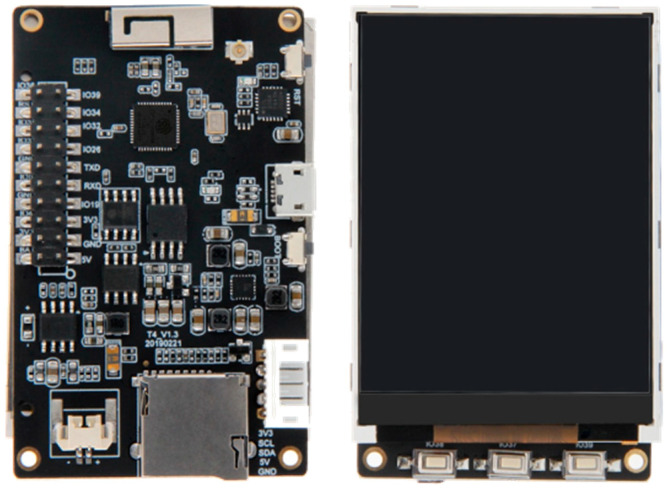
ESP-32 based module with integrated LCD.

PTL modules are based on an ESP-32 microcontroller with an integrated Wi-Fi interface (Table 1). The PTL also include an LCD, a user button and an enclosure (Figure 3). The modules can be powered using a USB or 5 V external power supply, and, in normal operation, consumes approximately 270 mA to 280 mA. The LCD has a diagonal of 56 mm and is connected to the ESP-32 controller using an I2C serial bus. The LCD screen is high contrast so that the information displayed on the screen is clearly visible.

**Figure 4:**
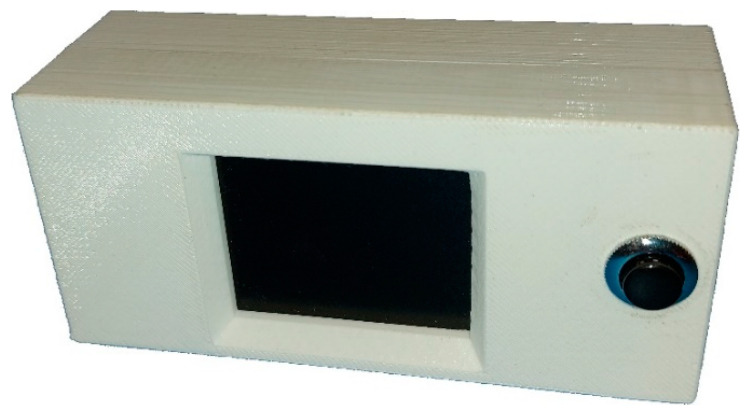
PTL module.

The enclosure of the PTL modules is made of a PLA (Polylactic acid) compound using a 3D printer (Figure 4). The external dimensions are 115 mm × 53 mm × 40 mm. The enclosure is small and compact and consists of two parts fixed together with screws. The enclosure has holes for the LCD and a pushbutton on the front side. Inside there are fittings that enable the attachment of a microcontroller with an LCD. On the rear side is a hole for the power cord and holes for mounting the adapters for the DIN rail mounting.

The modules are powered by a 220 V/5 V power supply unit. In the current set-up, 16 PTL modules are used, so the total power consumption is approximately 22.4 W. The PTL modules are connected to the power supply in parallel. Wiring is done inside the DIN rail (behind the PTL modules) using special connectors.

### 3.3. Software

#### 3.3.1. Main Application

**Figure 5:**
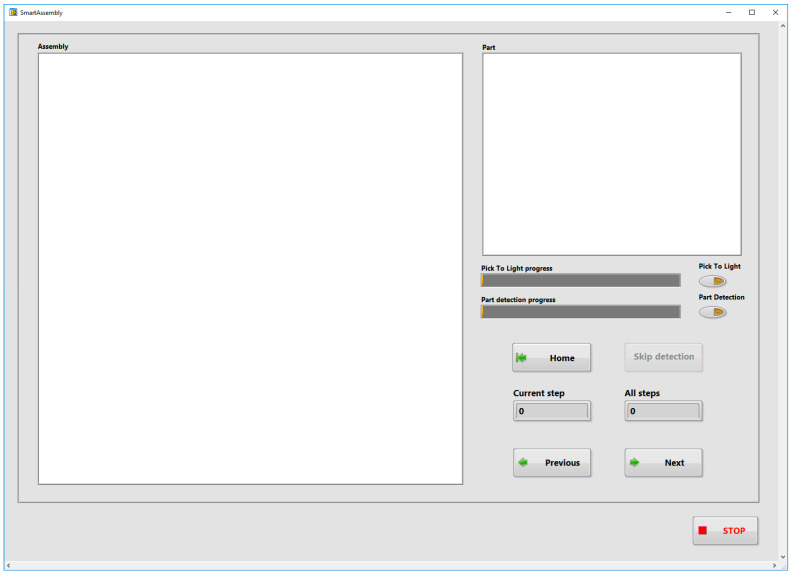
Main application GUI.

The main application guides the operator through the assembly process of the selected product. The program allows assembly product selection, the graphical display of the assembly steps, communication with the PTL modules and the object detection system, etc. The program has been designed using event structures and the state machine in the LabVIEW development environment. Events are related to the change of state of input objects (keys, selection objects, etc.) in the Graphical user Interface. The user interface (Figure 5) contains image display objects (Assembly, Part), user buttons (Home, Skip detection, Previous, Next), options objects (Pick To Light, Part detection) and various indicators (Pick 2 Light progress, Part detection progress, Current step, All steps).

**Figure 6:**
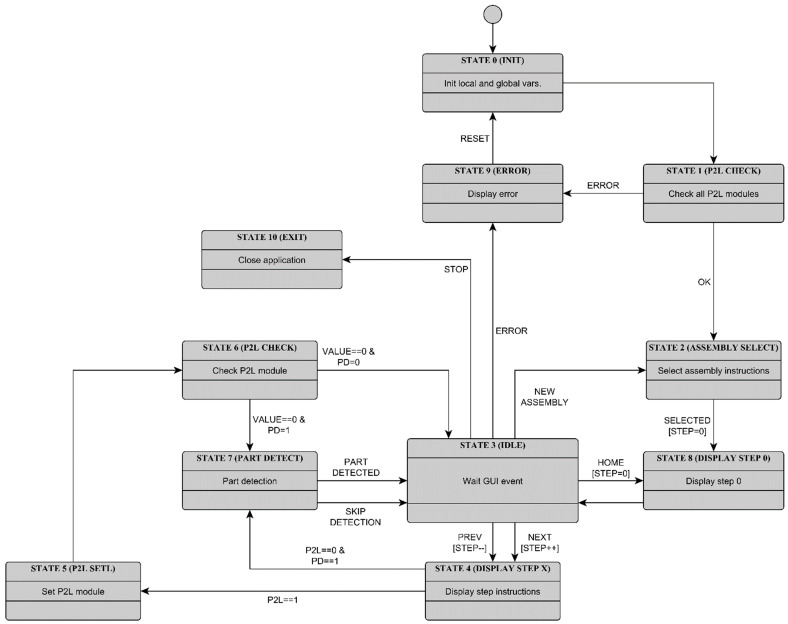
State diagram of the main application.

When the program is started, the local and global variables are initialized (State 0, Figure 6), and then all PTL modules are checked for presence and operation (State 1). The program then enters an idle state (State 3), where it waits for a user interface event. When the Next (Previous) button is pressed, the next (previous) assembly step is displayed (State 4). If the Home button is selected, the system returns to the initial assembly step (State 8).

In each assembly step, the main application shows the assembler the part currently needed for the assembly (Part object) and a picture showing where and how to place the component (Assembly object). The system allows enabling/disabling the use of the PTL modules (“Pick To Light” option) and object detection (“Part detection” option). If the first option (“Pick To Light”) is activated, the software provides the corresponding PTL module with information on how many parts the assembler has to take out of the associated bin (State 5). The PTL module displays the received value on the LCD, giving the assembler a visual indication of which bin contains the required components and how many are needed. The assembler must confirm each removal of a component from the bin by pressing a user button on the PTL module, which decreases the displayed value by one. In the meantime, the main application checks the state of the PTL module periodically (State 6). The algorithm enters the part detection state when the value equals 0, and the Part detection option is selected (“Part detection”, PD = 1). In this state, the assembler has to place the part within the camera field of view, and the part detection algorithm is triggered automatically. When the algorithm identifies the correct component or if the user skips object detection (“Part detection”, PD = 0), the algorithm returns to State 3 (Idle). The process is repeated until the assembler reaches the last assembly step (All steps).

The main application is designed to be universal. In the case of another product assembly, the application does not need to be modified. In this case, it is necessary to create a new folder with: (1) images of the individual parts, (2) a folder with images of the individual steps, and (3) an Excel document indicating which and how many components are needed in each assembly step. This Excel document contains three columns (Step number, PTL module number and Number of parts), and as many rows as the Number of assembly steps (Table 2).

#### 3.3.2. Communication Protocol

The implemented communication protocol is simple and currently allows two commands: (1) Setting the value on the LCD and (2) Checking the value displayed on the LCD. Each message contains three parts: (1) Command, (2) Data and (3) Message termination characters. Each message consists of 5 Bytes. The first one represents the command, the second and third contain the value, while the fourth and fifth represent termination characters (\r\n). When the PTL module receives a message, it parses the message and, depending on the command, performs the appropriate action (setting the value or checking the LCD value), then creates a return message and transmits it to the main application.

#### 3.3.3. Pick-To-Light Firmware

**Figure 7:**
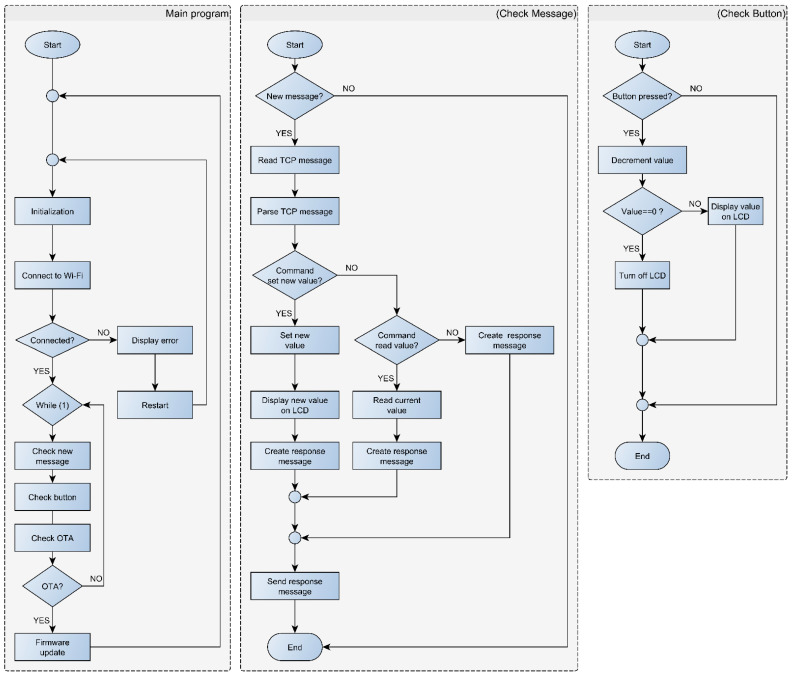
PTL module firmware flow chart.

At PTL module startup, the local and global variables are initialized (Figure 7). Then, the PTL module login into the selected Wi-Fi network with a preconfigured username and password. Since a large number of modules try to connect to the network at the same time, the connection may fail. In this case, the module is reset programmatically and tries to re-connect to the Wi-Fi network. The different initialization phases are shown in different colours on the LCD, to make it easier to identify a malfunctioning module. When the PTL module logs into the selected Wi-Fi network successfully, an IP address is assigned to the module. Since the IP address reservation is implemented on the router (according to the MAC address of the PTL module), the same IP address is assigned to the PTL module every time it starts up.

The algorithm then enters into an endless loop. An algorithm checks periodically if: (1) a new message has been received from the main application, (2) a key has been pressed on the PTL module, and (3) a request for a firmware upgrade has been received using OTA (Over-The-Air) technology. When a new command is received, the module parses the command, performs the requested action, generates a feedback message, and transmits the message back to the main application. In the case of a set message, it extracts the value from the received message and displays it on the LCD screen. In the case of a read message, the value displayed on the LCD is transferred to the sender (main application). When the button is pressed on the PTL module, the algorithm decrements the displayed value by one, and when the value equals 0, the LCD turns off.

The PTL modules implement OTA (Over-The-Air) technology. OTA allows the wireless transfer of new firmware to the selected PTL module. Using OTA, it is not necessary to connect the PTL module physically to a USB cable to update the firmware, but the update can be performed wirelessly using a Wi-Fi network. The new firmware can be downloaded using the development environment or an internet browser. The advantage of the latter approach is that the update can be performed even from a device that does not have the development environment installed. However, in the case of a firmware upgrade using the development environment, all PTL modules located on the local network appear in the development environment. The developer thus selects the desired PTL module and loads the new firmware on it.

#### 3.3.4. Object Detection

Object detection is a category of Computer Vision that deals with the recognition and location of objects in images. Object detection is divided based on the approach used: (1) machine learning approach or (2) deep learning approach. Machine learning mainly identifies certain features such as edges, colours, etc. These are then fed into a regression model that estimates the label and location of the object. In deep learning, however, the model itself learns what it is looking for. In our case, the latter approach has been used.

Deep learning-based object detection consists of two parts. The first part is the encoder, which runs the input image through several blocks and layers. These learn to extract statistical features used to find and label objects. The outputs from the encoder are then passed to the decoder, which predicts the “bounding boxes” and assigns to each of these a label of the class to which it estimates that the labelled object belongs (person, car, cyclist, etc.). Detection time is crucial when using object detection algorithms. The time depends mainly on the complexity and the computation the computer has to perform to get the desired result. Consequently, various algorithms have evolved that try to speed up the detection process as much as possible. Among the more well-known algorithms are (1) R-CNN (Region Convolutional Neural Network), (2) Fast R-CNN, (3) Faster R-CNN, (4) CenterNet, (5) YOLO (You Only Look Once), etc. YOLO algorithm has been used in presented project. YOLO is one of the market’s fastest and most widely used object detection algorithms, suitable for real-time object detection. The detection speed varies depending on the hardware and input data size (e.g., on graphics cards, object detection is 10+ times faster than on computer processors).

**Figure 8:**
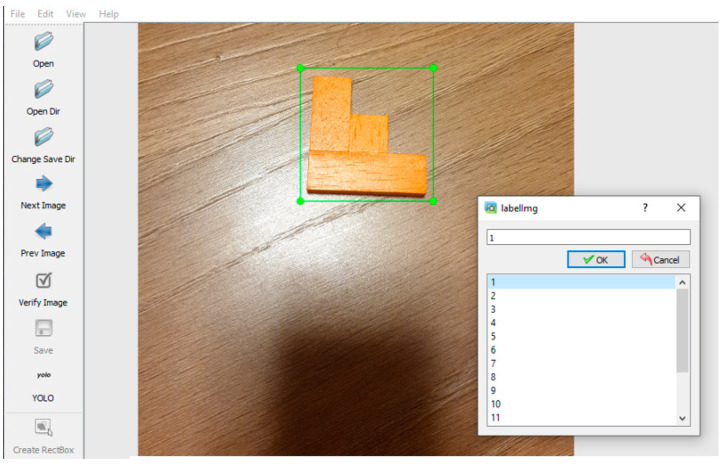
Object labelling using LabelImg tool.

Neural network learning requires a set of input data. Roughly speaking, the more inputs a network has, the better it learns. In the presented solution, the hardware equipment was not state-of-the-art; therefore, we used relatively small amounts of data, but enough to make the learned network usable. A neural network needs a “ground truth” to recognize which features it needs to learn. Ground truth is given as bounding boxes on the input images using two approaches. The most accurate is the manual labelling process, as this gives direct control over the accuracy of the frames. The other way is using roughly trained algorithms to recognize features and predict what is in the image based on what we have chosen to label. The labelling process is much faster but usually less accurate in this case. There is no guarantee that the algorithm will find all the objects in the image, and the frames are approximate in many cases. We took the first approach and manually labelled all the images using the “LabelImg” tool (Figure 8). For each object, about 500 images were captured from different angles, with different objects in the background, partially overlapped by other objects, etc. Each image was manually labelled with the objects to be searched and assigned with the appropriate identification number (ID).

The labelled images were then fed into a neural network that learned to recognize the objects it was looking for. A cloud service, “Google Colab”, was used for this purpose.

The image capture and object detection algorithm using a neural network are implemented in Python, using the neural network weights obtained from Google Colab. Communication with the main application is implemented as master/slave using TCP/IP protocol. The main application sends the ID of the object it wants to detect. The detection algorithm decodes the information accordingly, captures the image using a USB camera, performs the part detection and sends the detection result to the main application: (1) value 0 if the wrong object is detected or (2) the object ID if the correct object is detected. During the detection phase, the main application periodically sends object check requests until the correct object is detected or the user cancels the object detection in the main application GUI.

## 4. Results

**Figure 9:**
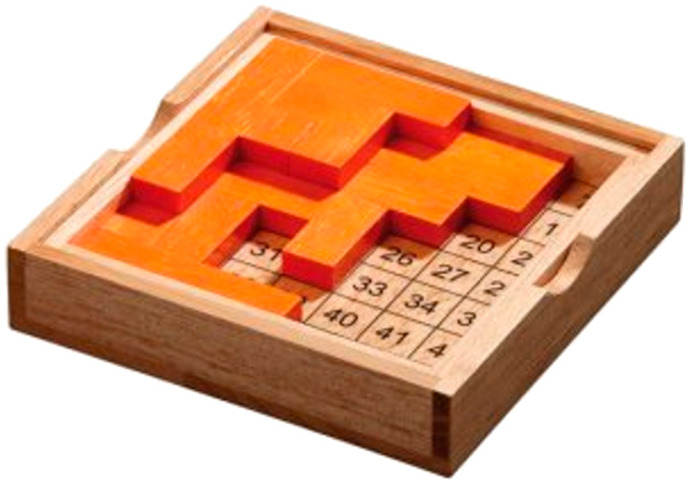
Gillux-Puzzle.

**Figure 10:**
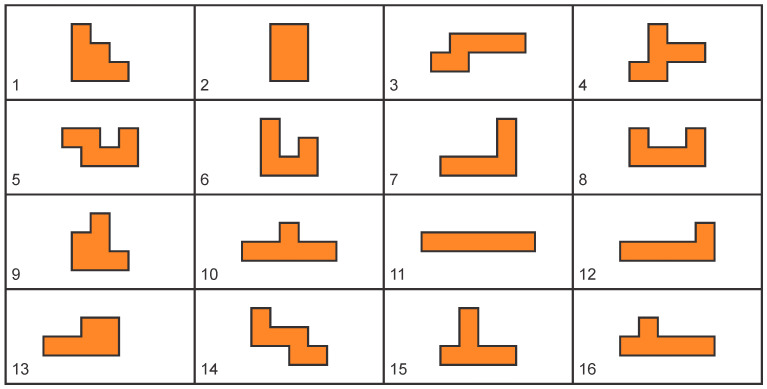
Gillux-Puzzle basic parts.

The system was tested using Gillux-Puzzle (Figure 9) mind game. Gillux-Puzzle is a mind game that includes 16 wooden parts (Figure 10) and a baseboard with 49 fields. The parts must be stacked on the base board in two levels so that one field (between 1 and 49) remains visible. There are 49 different possible solutions. Without graphical instructions, this mind game is quite challenging to assemble.

Although the presented system is designed for the guided assembly of industrial products, the test users were not assembling an industrial product but a mind game. The assembly process of a mind game does not require specific personal skills and tools, as is usually the case with industrial products; therefore, results between the test users are more comparable. We tested the presented system to identify differences in assembly times using original instructions and a guided system with and without Pick-to-Light technology. The test users were not professional assemblers but students at our faculty. They represent a novice assembler who has not yet acquired specific assembly skills.

**Figure 11:**
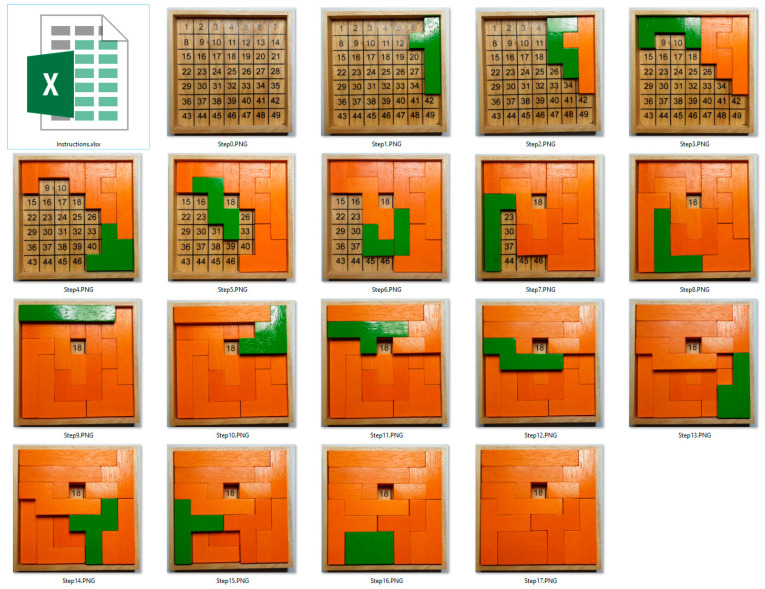
Gillux-Puzzle assembly steps (for solution field 18).

Figure 11 shows the process of solving the Gillux-Puzzle for field 18. The new part is displayed in green in each assembly step, while all previous ones are in orange. The green part is the one the assembler must take out of the corresponding bin in each assembly step and place correctly on the baseboard.

**Figure 12:**
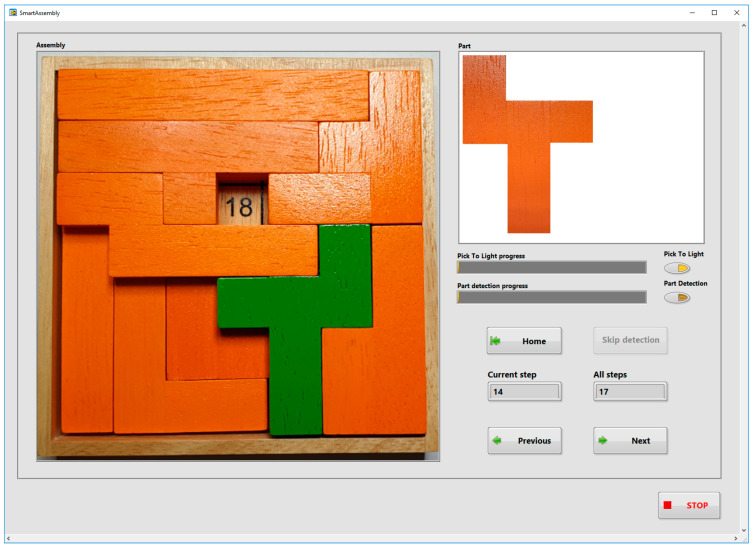
Main application GUI (Gillux-Puzzle, solution field 18, step 14/17).

**Figure 13:**
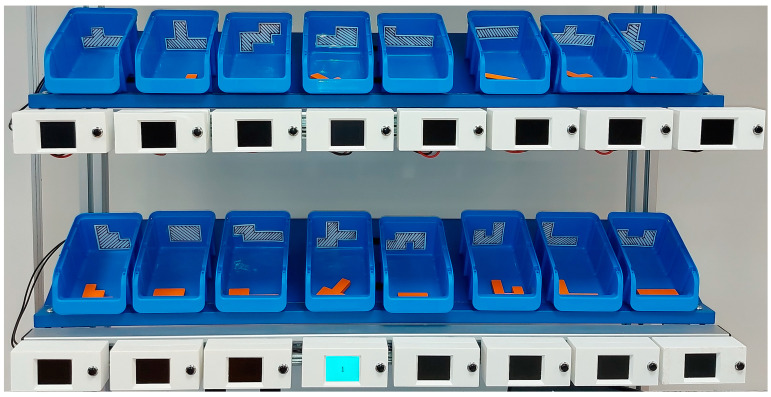
PTL modules (Gillux-Puzzle, solution field 18, step 14/17).

**Figure 14:**
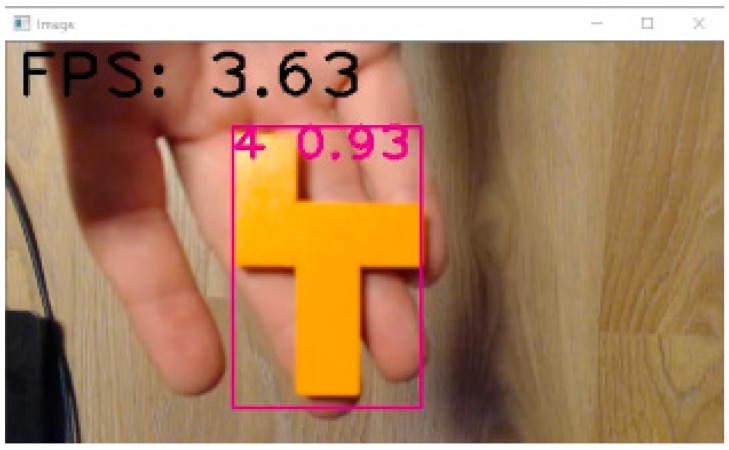
Part detection (Gillux-Puzzle, solution field 18, step 14/17).

Figure 12 shows the GUI seen by the assembler during the assembly of the Gillux-Puzzle. Step number 14 (out of 17) is shown. The “Part” field shows the part needed by the assembler in the current step. If the option “Pick To Light module” is enabled, then the main application sends the information to the corresponding PTL module (in this case, PTL module number 4), and the LCD of this module displays the value 1 (Figure 13). The user has to remove one part from the associated bin and confirm the removal by pressing a button on the PTL module. The part detection process is then started (if the “Part detection” option is enabled). The main application enables the Next (Previous) button when the correct part is detected (Figure 14), allowing the assembler to move to the next (previous) assembly step. In the presented example, part number 4 was detected (Figure 14) during the detection phase.

Table 3 shows the content of the Excel document file located in the folder with the images of the assembly steps (Figure 11). The document links the individual assembly steps with PTL modules.

### 4.1. Assembly Approaches

**Figure 15:**
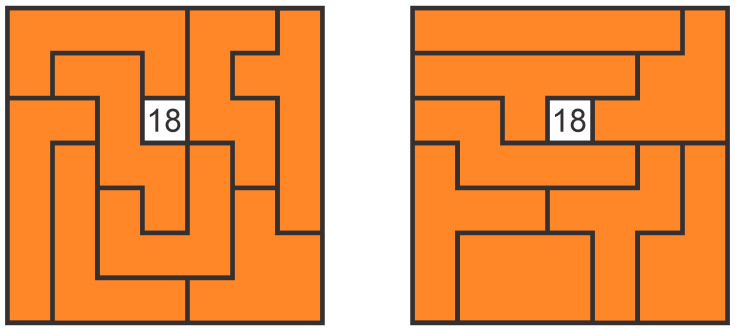
Original instructions for solution filed 18 (**left**—1st level, **right**—2nd level).

Eighteen volunteer assemblers (students) have tested the system. Each assembler carried out the following three assembly approaches:

1. The assembler has been issued the original instructions from the Gillux-Puzzle manufacturer. In this case, only the correct arrangement of parts in the 1st and 2nd levels is provided (Figure 15). The order of assembly in each level is not predefined and is, therefore, up to each user.
2. The assembler has been issued the guided instructions on the touchscreen monitor, but the PTL modules remain disabled. The assembly sequence is determined.
3. The assembler has been issued the guided instructions, shown on the touchscreen monitor, along with the PTL modules.

Each assembler performed three repetitions of the stated assembly approaches.

### 4.2. Results Using Original Instructions (OI)

**Figure 16:**
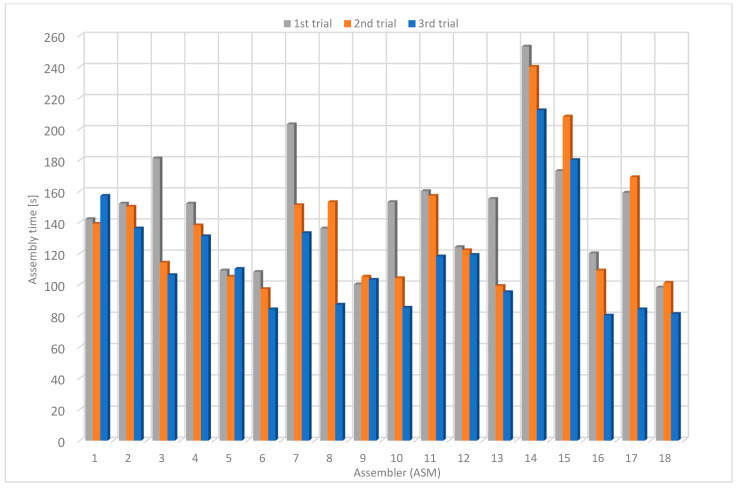
Assembly time using original instructions.

On average, the assemblers required 149 s on the first trial, 137 s on the second trial and 117 s on the third one (Table 4). The average assembly time was 134 s. The average assembly time was reduced by about 22 % from the first to the third attempt. The highest measured assembly time was 253 s, and the lowest was 80 s. On the third trial, the average assembly time was 117 s, the maximum 212 s and the minimum 80 s (Figure 16).

### 4.3. Results Using Guided Instructions without P2L Modules (P2L w/o P2L)

**Figure 17:**
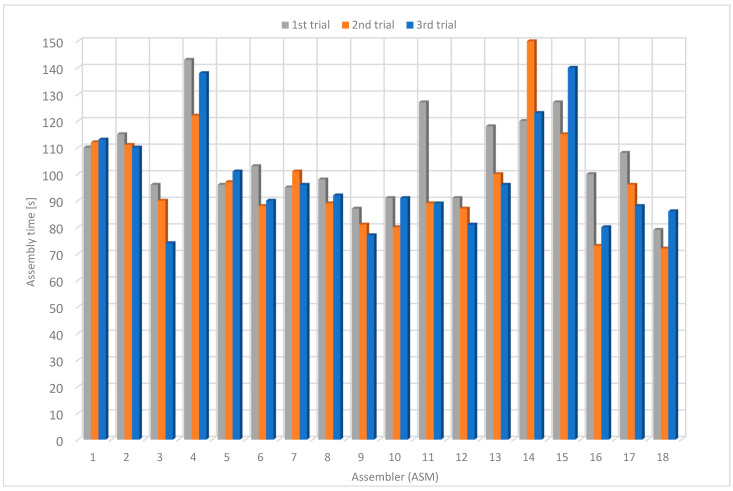
Assembly time using guided instructions without PTL modules.

Using the guided instructions without P2L modules, the average assembly time in the first trial was 106 s, in the second one 97 s and in the third one 98 s (Table 5). The average assembly time was 100 s. The assembly time decreased by approximately 7% from the first to the third trial. In the last trial, the maximum assembly time was 140 s, the minimum was 74 s, and the average was 98 s (Figure 17). Compared to the original instructions, the average assembly time was about 25% shorter, while the assembly time in the third (last) experiment was about 16% shorter.

### 4.4. Results Using Guided Instructions with P2L Modules (GI w/P2L)

**Figure 18:**
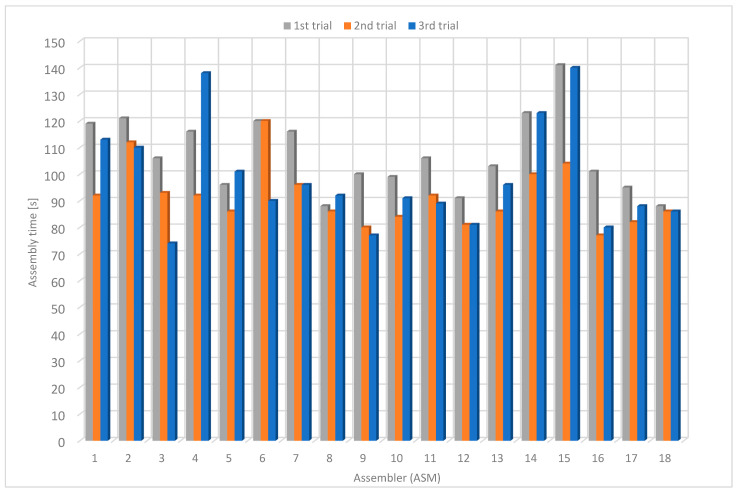
Assembly time using guided instructions with PTL modules.

Using the guided instructions with P2L modules, the average assembly time for the first trial was 107 s, for the second trial 92 s and for the third trial 88 s (Table 6). The average assembly time was 96 s. The assembly time decreased by about 18% from the first to the third trial. The maximum assembly time was 115 s, and the minimum was 81 s (Figure 18). Compared to the original instructions, the average time is shorter by about 29%, while the assembly time in the third (last) trial is shorter by about 25%. Compared to guided instructions without P2L modules, the average assembly time is shorter by about 5%, while the assembly time in the third trial is shorter by approximately 10%.

### 4.5. Comparison of Average Assembly Times

**Figure 19:**
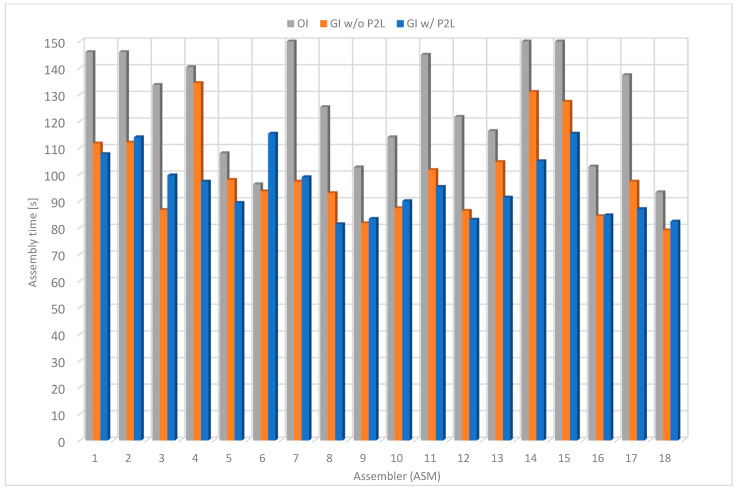
Average assembly time.

The average assembly times using the described methods are presented in Figure 19. The average time using the first method (OI) is 134 s, using the second one (GI w/o P2L) is 100 s and using the third one (GI w/o P2L) is 96 s. The results show that the lowest product assembly times were achieved using P2L modules. Most assemblers achieve a much shorter assembly time using the P2L method than using original instructions. Using the P2L modules, the assembly time was up to 30% shorter than using the original instructions and up to 10% shorter than using the guided instructions without the P2L modules.

The results show that, in general, the assembly time decreases with repetition. With each attempt, the user acquires specific skills, which are put into practice in the next attempt. In most cases, the average assembly time using the third procedure is shorter than the second one.

The assemblers reported that they felt uncomfortable using the first method and felt time pressure to assemble the product as quickly as possible. In addition, the users tried to assemble the puzzle in different ways, as reflected by the different assembly times. Due to the similarity between objects, the workers sometimes took out the wrong pieces, which generated additional time delays.

Similar issues were not detected by using the guided assembly workstation, which is designed to prevent such events. The workers felt more secure and self-confident, and their stress levels were much lower due to reassurance during the assembly process. Although the assembly sequence is well-defined, there are delays in finding the parts. However, the time to find parts is reduced by repetition, as users eventually remember where each assembly part is located.

The third procedure is the fastest, although there is no significant time difference compared to the second procedure. The system directs the assembler to find the required assembly part quickly. However, the assemblers reported that the assembly time increased as they often forgot to confirm the removal of the part on the PTL module. They realized this when they wanted to move to the next assembly step, which was impossible since the Next button was disabled.

## 5. Conclusions

This paper presents a prototyping system that enables guided assembly of new products using pick-to-light technology. The presented solution demonstrates that the technology, used primarily in warehouse systems, can also be applied usefully in the product assembly process. The presented system is useful in the case of the assembly process of complex products containing a large number of components. Although only 16 modules were used in the presented example, the system is scalable, and can easily be upgraded to a larger number of modules. Although the example presented does not illustrate the process of assembling an industrial product, the mind game is an excellent example of a product that is difficult to assemble without graphical instructions or prior practice. The main application is designed to be universal. In the case of another product assembly, the application does not need to be modified; just a few files and images have to be added to the appropriate folder. This is only the case when computer vision is not used. However, if computer vision is used, the process is much more complex, as many images have to be captured for each part, then the parts have to be manually labelled in the images, and finally, the neural network has to be trained.

The Assembly process using PTL modules is faster than the conventional approach since PTL modules direct the user quickly to the bin where the necessary components are placed. Part detection is useful in the first assembly of a product to check if the correct parts are placed into the bins; however, it is slightly less useful later in the assembly process since it slows down the assembly process and can therefore be skipped. Parts detection is much more useful in the inverse process, i.e., in a product’s disassembly process and filling the bins with parts. This part has not yet been implemented but is planned for the near future. During the system testing phase, we realized that the assembly procedure using PTL technology, but without removal confirmation, would be beneficial. We will add this option in an updated version of the main application.

In addition, the firmware of the PTL modules is planned to be modified. In the current version, the Wi-Fi network to which the PTL module connects is fixed in advance. In the upgraded version, the Wi-Fi network will be configurable using the web interface. Each PTL module will create an access point and web page through which it will be possible to select the network to which the PTL module should connect and set a password to access the selected network.

## Figures and Tables

**Table 1:** ESP-32 specifications.

Specification	Value
MCU	Xtensa Dual-Core 32 bit LX6, 600 SMIPS
Typical frequency	160 MHz
GPIO	36
ADC	12 bit
SPI/I2C/I2S/UART	4/2/2/2
Flash	16 MB
SRAM	512 kB
Wi-Fi	integrated, 2.4 GHz

**Table 2:** Example of the content of an Excel file.

Step Number	PTL Module Number	Number of Parts
0	0	0
1	3	1
2	7	1
3	6	1

**Table 3:** The content of Instructions.xlsx document for assembly of Gillux-Puzzle (solution field 18).

Step Number	PTL Module Number	Number of Parts
0	0	0
1	16	1
2	5	1
3	18	1
4	9	1
5	14	1
6	6	1
7	12	1
8	7	1
9	11	1
10	1	1
11	10	1
12	3	1
13	13	1
14	4	1
15	15	1
16	2	1
17	0	0

**Table 4:** Assembly time using original instructions.

Assembler	1st Trial [s]	2nd Trial [s]	3rd Trial [s]	Average Time [s]
1	142	139	157	146.0
2	152	150	136	146.0
3	181	114	106	133.7
4	152	138	131	140.3
5	109	105	110	108.0
6	108	97	84	96.3
7	203	151	133	162.3
8	136	153	87	125.3
9	100	105	103	102.7
10	153	104	85	114.0
11	160	157	118	145.0
12	124	122	119	121.7
13	155	99	95	116.3
14	253	240	212	235.0
15	173	208	180	187.0
16	120	109	80	103.0
17	159	169	84	137.3
18	98	101	81	93.3

**Table 5:** Assembly time using guided instructions without PTL modules.

Assembler	1st Trial [s]	2nd Trial [s]	3rd Trial [s]	Average Time [s]
1	110	112	113	111.7
2	115	111	110	112.0
3	96	90	74	86.7
4	143	122	138	134.3
5	96	97	101	98.0
6	103	88	90	93.7
7	95	101	96	97.3
8	98	89	92	93.0
9	87	81	77	81.7
10	91	80	91	87.3
11	127	89	89	101.7
12	91	87	81	86.3
13	118	100	96	104.7
14	120	150	123	131.0
15	127	115	140	127.3
16	100	73	80	84.3
17	108	96	88	97.3
18	79	72	86	79.0

**Table 6:** Assembly time using guided instructions with P2L modules.

Assembler	1st Trial [s]	2nd Trial [s]	3rd Trial [s]	Average Time [s]
1	119	92	112	107.7
2	121	113	109	114.0
3	106	93	100	99.7
4	116	92	84	97.3
5	96	86	86	89.3
6	120	120	106	115.3
7	116	96	85	99.0
8	88	86	70	81.3
9	100	80	70	83.3
10	99	84	87	90.0
11	106	92	88	95.3
12	91	81	77	83.0
13	103	86	85	91.3
14	123	100	92	105.0
15	141	104	101	115.3
16	101	77	76	84.7
17	95	82	84	87.0
18	88	86	73	82.3

## Data Availability

Not applicable.
